# The Emerging Role of the IL-17B/IL-17RB Pathway in Cancer

**DOI:** 10.3389/fimmu.2020.00718

**Published:** 2020-04-21

**Authors:** Jérémy Bastid, Cécile Dejou, Aurélie Docquier, Nathalie Bonnefoy

**Affiliations:** ^1^OREGA Biotech, Ecully, France; ^2^IRCM, Institut de Recherche en Cancérologie de Montpellier, INSERM U1194, Université de Montpellier, Institut Régional du Cancer de Montpellier, Montpellier, France

**Keywords:** IL-17, IL-17B, IL-17RB, inflammation, cancer, cancer therapy

## Abstract

Among inflammatory mediators, a growing body of evidence emphasizes the contribution of the interleukin 17 (IL-17) cytokine family in malignant diseases. Besides IL-17A, the prototypic member of the IL-17 family, several experimental findings strongly support the role of the IL-17B/IL-17 receptor B (IL-17RB) pathway in tumorigenesis and resistance to anticancer therapies. In mouse models, IL-17B signaling through IL-17RB directly promotes cancer cell survival, proliferation, and migration, and induces resistance to conventional chemotherapeutic agents. Importantly, recent work by our and other laboratories showed that IL-17B signaling dramatically alters the tumor microenvironment by promoting chemokine and cytokine secretion which foster tumor progression. Moreover, the finding that elevated IL-17B is associated with poor prognosis in patients with pancreatic, gastric, lung, and breast cancer strengthens the results obtained in pre-clinical studies and highlights its clinical relevance. Here, we review the current understanding on the IL-17B/IL-17RB expression patterns and biological activities in cancer and highlight issues that remain to be addressed to better characterize IL-17B and its receptor as potential targets for enhancing the effectiveness of the existing cancer therapies.

## Introduction

The IL-17 cytokine family and its receptors play crucial roles in normal host immune responses. Their dysregulated expression has been associated with many human diseases, notably inflammation and cancer. The IL-17 family includes six members (IL-17A to IL-17F) with different sequence homology and functions (1). These cytokines exert their activities through binding to IL-17 receptors (IL-17R, IL-17RA to IL-17RE) that function as homo- or heterodimeric complexes. IL-17A is the prototypic member of the IL-17 family and is predominantly produced by T helper 17 (Th17) cells. IL17A binding to IL-17RA/IL17RC heterodimers leads to the production of cytokines and chemokines, such as tumor necrosis factor a (TNF-α), IL-6, CXCL8, and CXCL1, involved in mechanisms of the host defense against extracellular bacterial and fungal infections (2, 3). However, IL-17A overproduction has been associated with chronic inflammatory disorders, autoimmune diseases and cancer (2, 4–6). Among other members of the IL-17 family, IL-17B was originally described as increased during intestinal inflammation (7). Moreover, it stimulates TNF-α and IL-1β production by the human monocytic leukemia THP-1 cells (7) and promotes neutrophil migration upon intraperitoneal administration, suggesting a pro-inflammatory role (8). More recent findings strongly suggest a role for the IL-17B/IL-17RB pathway in tumorigenesis. For instance, in mouse models, IL-17B signaling through IL-17RB promotes cancer cell survival, proliferation, and migration (9–12), while in humans, elevated IL-17B expression has been associated with poor prognosis in patients with different cancer types (10–12).

In this review, we summarize the knowledge on the expression and biological activities of the IL-17B cytokine and its receptor, and then focus on their implication in tumorigenesis highlighting gaps that remain in our understanding of this topic.

## IL-17B and its Receptor IL-17RB

### IL-17B Expression

Following the discovery of IL-17A, which was originally named CTLA8, screens to identify homologous genes led to the discovery of the IL-17B, IL-17C, IL-17D, IL-17E (known as IL-25), and IL-17F cytokines. Human IL-17B was cloned in 2000 by homology-based screening of an expressed sequence tag database, followed by amplification from a fetal tissue cDNA library (7, 8). The IL-17B protein shares 88% of homology with its murine ortholog but only 29% homology with human IL-17A (8). IL-17B is secreted as a non-covalent dimer glycoprotein consisting of 180 amino acids and has a predicted molecular mass of 20.4 kDa as a monomer (2, 13). The human *IL-17B* gene was mapped to chromosome 5q32–34, and its mRNA is strongly expressed in adult pancreas, small intestine, stomach, testis and more weakly in spinal cord, prostate, colon and ovary (7, 8). IL-17B expression was also detected in rheumatoid synovial tissues from patients with rheumatic arthritis, where it is mainly produced by neutrophils (14), as well as in chondrocytes (15) neurons (16) and naive, memory and germinal center B cells (17). Importantly, the IL-17B and IL-17A expression profiles are very different. Indeed, IL-17B was never detected in activated CD4 T cells, particularly Th17 CD4 T cells that are the main IL-17A source (7).

### IL-17B Receptor Expression

IL-17B binds to its receptor IL-17RB, a 47.9 kDa transmembrane protein (462 aa) that belongs to the IL-17 receptor family. IL-17RB has a SEFIR cytoplasmic domain implicated in homotypic dimerization and recruitment of signaling proteins (11, 18) (shared with IL-17RA) and a TRAF6-binding domain (not found in IL-17RA). IL-17B shares its receptor IL-17RB with IL-17E (also known as IL-25) that binds to the heterodimeric IL-17RA/IL-17RB complex (19). The binding affinity (KD) of IL-17B for IL-17RB is around 30-fold lower than that of IL-17E, with a similar association rate (Kon) but a substantially faster dissociation rate (Koff) (20).

IL-17RB is expressed in various endocrine tissues and in epithelial cells in different organs such as kidney and liver and mucosal tissues (8, 19, 21). Elevated IL-17RB expression is also found lung tissues from asthmatic patients and in skin lesions from patients with atopic dermatitis (22). IL-17RB expression in human innate type 2 lymphocytes, natural killer T (NKT) cells, and Th2 cells (20, 22) suggests a potential role in immune cells. In these human cells IL-17B promotes IL-33-driven type 2 immune responses, a function shared with IL-17E, but not with IL-17A (20).

### IL-17RB Signaling Pathway

Data on the IL-17RB signaling pathway are limited and mainly described after binding of IL-17E. Upon ligand binding, IL-17RB activates the canonical NK-κB pathway as well as ERK, JNK, and p38 (19, 23, 24). Moreover, TRAF6 binds to IL-17RB independently of its ligand and participates in IL-17RB-dependent NF-κB activation (23).

### IL17B/RB Pathway in Inflammatory Diseases

IL-17B was originally described as a proinflammatory cytokine (8, 9). Indeed, IL-17B is strongly expressed in the paws of arthritic mice and administration of a polyclonal anti-IL-17B antibody ameliorates collagen-induced arthritis in these mice (25). Moreover, IL-17B has been detected in rheumatoid synovial tissues from patients with rheumatic arthritis. In these tissues, IL-17B is produced by neutrophils and potentiates TNF-α effect on the production of cytokines and chemokines, such as IL-6, G-CSF, and CCL20, known to control immune cell trafficking to inflamed tissues (14). Interestingly, although IL-17B and IL-17E (IL-25) share a common receptor, IL-17RB, IL-17B, and IL-17E deficiency lead to opposite results in a model of acute colitis induced by dextran sulfate sodium. These results indicate that IL-17E has a pathogenic role in colon inflammation, whereas Il-17B has a protective role. Moreover, IL-17B inhibits IL-17E binding to IL-17RA– IL-17RB complexes on epithelial cells, and limits IL-17E-induced IL-6 production by colon epithelial cells (26). Altogether, these findings suggest that if both cytokines are concomitantly produce at the same site, IL-17B might restrict IL-17E/IL-17RB signaling. The two cytokines have opposite roles also in *Citrobacter rodentium* infection and allergic asthma (26). Similarly, in murine cancer models and patients, IL-17B exhibits protumor roles and IL-17E antitumor activities (see just below).

## IL-17B/IL-17RB Pathway in Tumors

### Expression and Prognosis

In the last decade, several reports highlighted the potential role of the IL-17B/IL-RB pathway in cancer (9–39). High expression of IL-17B or its receptor has been associated with poor patient prognosis in different cancer types (see Table 1). For instance, in a cohort of 69 patients with ductal invasive breast carcinoma, Furuta et al., were the first to show that an IL-17RB (referred to as IL-25R in this study) was upregulated in 19% of patients. Moreover, IL-17RB detection was significantly correlated with poor prognosis and high mortality rate in this cohort (27). These first results were then confirmed by Huang et al., in an independent cohort of 179 patients with breast cancer (28). In this study, the correlation between IL-17RB expression and poor prognosis was statistically significant even after adjustment for several clinical parameters (age, tumor size, lymph node status and estrogen receptor expression). The authors also observed that IL-17RB expression was associated with HER2 amplification and survival rate was lowest in patients with high expression of both IL-17RB and HER2 (28). Finally, in another cohort of 143 patients, we showed that not only IL-17RB but also IL-17B expression is associated with reduced patient survival. Then, we analyzed microarray data of 1809 patients with breast cancer, and found that high IL-17B expression was significantly correlated with poorer prognosis in the whole population and in the basal-like subtype, but not in other breast cancer subtypes. Conversely, IL-17A expression was associated with favorable outcomes in the whole population and in the different molecular subtypes from this cohort (10).

**Table 1:** IL-17B IL-17RB expression in cancers.

**Tumor**	**Expression—Prognosis**	**Mechanism—Models**	**References**
Breast	IL-17RB upregulation is correlated with poor prognosis.	ShRNA-dependent reduction of IL-17B decreases tumor growth and invasiveness of MDA-MB468 human breast cancer cells.	(27)
Breast	IL-17RB upregulation is correlated with poor prong/osis.	IL-17RB recruits TRAF6, activates NF-kB, upregulates Bcl-2, and induces resistance to etoposide. IL-17RB or IL-17B targeting with Abs attenuates human MDA-MB361 breast cancer cell colony formation *in vitro* and tumor growth *in vivo*.	(28)
Breast	High expression of IL-17B and IL-17RB is associated with poor prognosis. IL-17B upregulation is associated with poorer survival in patients with basal-like breast cancer.	MCF7 and MDA-MB468 human breast cancer cells that overexpress IL-17B are resistant to paclitaxel. Treatment with anti-IL-17RB antibodies restores breast tumor chemosensitivity *in vivo*.	(10)
Breast		TGF-β secreted by Treg cells up-regulates IL-17RB on 4T1 and EMT6 murine breast cancer cells via Smad2/3/4 signaling and increases their tumor growth and metastatic potential *in vivo*.	(29)
Pancreas	IL-17RB overexpression is associated with metastasis and poor clinical outcome.	Depletion of IL-17B or IL-17RB by shRNA or treatment with anti-IL-17B or anti-IL-17RB antibodies reduces CFPAC-1 and BxPC3 pancreatic cell line colony formation, invasion, tumor growth, and metastasis in xenograft models.	(11)
Gastric	IL-17RB expression in group 2 innate lymphoid cells (ILC2) is higher in peripheral blood mononuclear cells from patients with gastric cancer than in healthy donors.	IL-17RB expression by ILC2.	(30)
Gastric	Overexpression of IL-17RB correlates with poor prognosis. IL-17B level in serum is higher in patients with gastric cancer than in healthy donors.	IL-17B activates the AKT/β-catenin pathway and promotes stemness and EMT of MGC-803 human gastric cancer cells.	(31)
Glioblastoma	A signature with 6 enriched cytokines (incl. enriched expression of IL-17B) predicts poor overall survival		(32)
Primary testicular lymphoma	A signature with 25 enriched cytokines (including IL-17B) predicts poor survival.		(33)
Colon	IL-17B expression is increased in moderate and poorly differentiated tumors.	IL-17RB is expressed by colon epithelial cells. Neutrophils are the main source of IL-17B in the stroma.	(34)
Prostate	IL-17RB expression is higher in cancer-associated fibroblasts from prostate cancer patients than in fibroblasts from benign prostate hyperplasia.	IL-17RB expression by cancer-associated fibroblasts.	(35)
ATL	Overexpression of IL-17RB in leukemic cells.	Tax induces IL-17RB expression in a NF-kB dependent-manner in the HTLV-1 transformed T cell lines C8166 and MT-2.	(36)
Thyroid	IL-17RB is upregulated in thyroid cancer tissues compared with normal thyroid tissues.	IL-17B/IL-17RB signaling induces ERK activation, MMP-9 expression and promotes migration and invasion of SW1736 thyroid cancer cells. IL-17RB signaling contributes to tumor growth and metastasis formation of SW1736 tumor cell xenografts.	(37)
Lung	High IL-17B expression is associated with poor overall survival. High IL-17RB expression is associated with positive lymph nodes and distant metastases and positive distant metastases, and is predictive of disease-free survival and overall survival.	IL-17RB expression positively correlates with the invasion potential of lung cancer cell lines. IL-17RB promotes invasion/migration of H441 lung carcinoma cells through activation of the ERK signaling pathway, and its overexpression increases their metastatic potential *in vivo*.	(12)
AML	IL-17B and IL-17RB mRNA expression is significantly upregulated in patients with AML.	IL-17B/IL-17RB signaling drives MOLM-13 AML cell resistance to Ara-C (ERK/NF-kB/Bcl-2). Ara-C increases IL-17B expression.	(38)

Besides breast cancer, Wu et al., showed that in a cohort of 111 patients with pancreatic cancer, high expression of IL-17RB expression strongly correlates with poor differentiation, metastasis, and tumor stage using the TNM staging system. They found that in patients with high IL-17RB expression, prognosis is worse and malignancy is enhanced (11). More recently, high IL-17RB expression was correlated with poor prognosis also in patients with gastric cancer, where the percentage of IL-17RB positive cancer cells is high in grade II to IV tumors and low in grade I tumors (31). In lung cancer, microarray dataset analysis also associated *IL-17B* and *IL-17RB* gene expression with poor patient survival. Moreover, immunohistochemistry analysis also showed that IL-17RB is up-regulated in patients with lung adenocarcinoma compared with normal lung tissues specimens and is associated with lymph node and distant metastasis as well as reduced progression-free-survival and overall survival (12). Finally, by ranking cytokine-encoding genes based on their survival predictive values in the Chinese Glioma Genome Atlas database (*n* = 105 patients), Cai et al., identified *IL-17B* as one of the six enriched genes (among 593) with the strongest predictive value for a poor overall survival in patients with primary glioblastoma (32).

Besides solid tumors, analysis of the profiling data of 730 immune response genes in 60 primary testicular lymphomas obtained with the Nanostring technology recently identified a 25-gene signature that characterizes patients with the shortest 5-year progression free survival. This signature is enriched in cytokines and cytokine receptors and includes IL-17B (33). Additionally, mRNA expression profiles analysis in the HemaExplorer database showed that IL-17B and IL-17RB are strongly expressed in acute myeloid leukemia (AML), compared with normal hematopoietic stem cells. In line with the *in-silico* analysis results, IL-17B, and IL-17RB mRNA and protein expression were significantly increased in AML blasts compared with cells from healthy controls. Particularly, their expression was dramatically increased in bone marrow supernatant from patients with AML compared with healthy donors (ELISA analysis) (38).

Thus, by combining bioinformatics analysis of gene expression datasets and protein analysis in several independent cohorts, these studies clearly show the association between a deregulated expression of the IL-17 and/or IL-17RB and poor prognosis in many different cancers. The IL-17B/IL-17RB signaling pathway role in tumorigenesis and treatment resistance is mediated through different mechanisms, not fully understood yet, as discussed in the next paragraph.

## Mechanisms of Action

**Figure 1:**
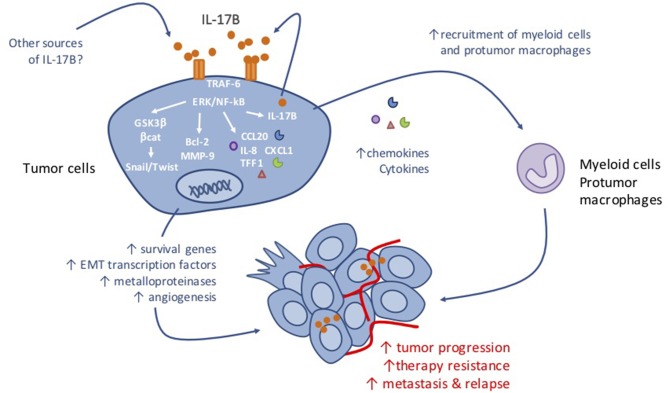
Anticipated mechanism of action of IL-17B in tumorigenesis.

The IL-17B/IL-17RB pathway is considered as a signaling cascade that promotes cancer cell survival, proliferation and migration. The pro-tumor functions associated with the IL-17B/IL-17RB pathway are diverse and complex because they involve mechanisms that act directly on tumor cells, and also indirect mechanisms that lead to tumor microenvironment remodeling (see Table 1 and Figure 1).

*In vivo* mouse models and *in vitro* cell assays indicate that in different tumor cell types, IL-17B signaling is critical for tumorigenesis promoting cancer cell survival and proliferation. Mechanistic studies in the MDA-MB 361, MDA-MB468, and MCF-7 breast cancer cell lines revealed that IL-17B promotes breast cancer cells survival *in vitro* by activating the ERK and NF-kB pathways and by enhancing the expression of anti-apoptotic Bcl-2 family members (10, 28). This leads to resistance to chemotherapeutic drugs, such as etoposide (a topoisomerase II inhibitor) (28), and paclitaxel (a spindle poison) (10). Similar results were recently obtained in leukemic cells by Guo et al., who demonstrated that the IL-17RB pathway promotes the survival of MOLM-13 AML cells by increasing ERK and NF-KB phosphorylation and Bcl-2 level and consequently, resistance to the purine analog Ara-C, the frontline chemotherapeutic agent for AML (38). Importantly, in each study, inhibition of the IL-17B/IL-17RB axis by downregulating receptor expression in tumor cells or by using neutralizing anti-IL-17RB antibodies restored chemosensitivity *in vitro* (28, 38) and *in vivo* (10). Similarly, IL-17B or IL-17RB silencing in cancer cells or treatment with antibodies targeting IL-17RB reduced proliferation of MDA-MB361 breast cancer cells and MOLM-13 AML cells *in vitro* and tumor growth *in vivo* in xenograft models based on these cell lines (28, 38). Interestingly, IL-17RB knockdown in MOLM-13 AML cells had a stronger effect than IL-17B knockdown *in vivo*, reflecting the potential contribution of the microenvironment-derived IL-17B to the signal delivered to IL-17RB-positive leukemia cells. As a corollary to this observation Bie et al., recently showed that non-tumor tissue-derived IL-17B promotes the proliferation and migration of MGC-803 gastric cancer cells (31). This also suggests that stimulation by any other IL-17RB-positive cells from the tumor microenvironment might indirectly contribute to the tumor progression. Indeed, Bie and colleagues showed that mesenchymal stem cells (MSCs) produce IL-6, IL-8, TGF-β, and CCL-5 following IL-17B stimulation and that supernatants collected from MSCs incubated with recombinant IL-17B promote the proliferation of MGC-803 gastric cancer cells *in vitro* (9). Thus, both direct IL-17RB signaling in the tumor cells and indirect IL-17RB signaling in cells present in the tumor microenvironment, such as MSCs, might contribute to promote tumor proliferation.

In addition to the effect on cancer cell proliferation and survival, the IL-17B/IL-17RB signaling pathway induces stemness and epithelial to mesenchymal transition (EMT) of MGC-803 gastric cancer cells through activation of the AKT/GSK-3β/β-catenin pathway and the up-regulation of Sox2, Oct4, and Nanog proteins. The relevance of these *in vitro* results to human gastric cancer is supported by the positive correlation between *IL-17RB* and *OCT4, NANOG, LGR5*, and *SALL4* mRNA expression in human gastric cancer tissues (31). Interestingly, IL-17RB signaling through the ERK/GSK-3β/β-catenin pathway has been associated also with EMT in lung cancer. Indeed, Yang et al., recently demonstrated that in lung cancer cell lines, IL-17RB-mediated activation of the ERK pathway is critical to maintain the expression of Snail and Twist, two key transcription factors for EMT induction. Specifically, in A549 an CL1-5 lung cancer cell lines that spontaneously expressed high level of IL-17RB, Snail and Twist expression was decreased upon IL-17RB knockdown. These *in vitro* results were strengthened by immunohistochemistry analysis of a cohort of 139 primary lung tumors in which IL-17RB expression was positively correlated with Snail or Twist expression (12).

Activation of the ERK/GSK-3β/β-catenin pathway following IL-17RB stimulation also promotes the invasion and the migration of H441 and CL1-0 human lung cancer cells *in vitro*. This effect is lost by inhibiting ERK1/2 phosphorylation using the MEK/ERK inhibitor PD98059. Furthermore, the authors showed that IL-17RB overexpression in the H441 significantly increases in the number of metastatic nodules in the lung of xenografted mice. In patients with lung cancer, IL-17RB expression level correlates with lymph node and distant metastasis occurrence (12). These results connect the IL-17RB pathway to the control of metastasis formation, and support previous findings in thyroid (37) and pancreatic cancer (11). Indeed, in the SW1736 thyroid cancer cell line, IL-17B-dependent stimulation of IL-17RB induces ERK1/2 activation and increases expression of the matrix metalloproteinase MMP-9 expression, a key mediator of tumor invasion and metastasis formation. This results in an increased migration and invasion capacities of SW1736 cells both *in vitro* and *in vivo* (37). In pancreatic cancer, high IL-17RB expression has been associated with postoperative metastases in patients (11). Conversely, *IL-17RB or IL-17B* knockdown in CFPAC-1 and BxPC3 pancreatic cancer cell reduces their soft agar colony formation in soft agar and cell invasion *in vitro. In vivo* studies showed that tumor growth and metastasis formation are reduced in mice xenografted with *IL-17RB* or *IL-17B* knockdown cells compared with parental CFPAC-1 and BxPC3 cells. Similarly, treatment with an IL-17B neutralizing antibody showed reduced CFPAC-1 and BxPC3 tumor cell xenograft growth and metastasis formation *in vivo* (11). Interestingly in this study, Wu et al., found that the IL-17B/IL-17RB pathway supports tumorigenicity and metastasis formation of human pancreatic cancer cells through the activation of ERK1/2 signaling. This resulted in the expression of the pro-inflammatory cytokines and chemokines CCL20, CXCL1, IL-8, and TFF1 leading to the recruitment of macrophages in the tumor microenvironment and of vasculogenic endothelial cells to promote angiogenesis. In agreement, shRNA-mediated of CCL20, CXCL1, or TFF1 depletion in CFPAC-1 pancreatic cancer cells significantly decreased the percentage of macrophages that interact with tumor cells *in vivo*, while IL-8 depletion reduced CD31+ endothelial cell recruitment. Importantly, although TFF1 is predominantly expressed by cancer cells, the authors detected CCL20, CXCL1, and IL-8 in cancer cells and also in the surrounding stroma (11). Likewise, chemokines might also be secreted by tumor-infiltrating cells as a result of stimulation of IL-17RB-expressing stromal, and might contribute to macrophages and endothelial cell recruitment to promote cancer progression.

## Conclusions and Future Directions

Altogether, these studies clearly identified IL-17B and IL-17RB as key actors of cell tumorigenesis by enhancing the survival and the proliferative, migratory and invasive properties of tumor cells. Moreover, IL-17RB-mediated secretion of soluble factors will ultimately reshape the tumor microenvironment toward a macrophage-enriched infiltrate that might impair the anti-tumor immune response and favor resistance to treatments (Figure 1). In fact, our own unpublished data in mouse models suggest that IL-17B-driven alterations in the TME are the major contributors of the anticancer effect after IL-17B neutralization. Therefore, targeting Il-17B or its receptor might represent an interesting therapeutic option for cancer therapy. However, as IL-17RB is a common receptor for both IL-17B and IL-17E that binds to the heterodimeric complex IL-17RA and IL-17RB (19), the anticancer effect of IL-17E (IL-25) must be taken into account. Indeed, unlike IL-17B, IL-17E (or IL-25) causes caspase-mediated apoptosis of breast cancer cells and reduces colony formation of IL17RB-expressing breast tumor cell lines *in vitro* (28). Furthermore, IL-17E (IL-25) markedly reduces growth of MDA-MB468 breast tumor xenografts *in vivo*, while IL-17B increases it (27). These results suggest that in cancer, like in mucosal inflammation (26) IL-17B and IL-17E might have opposite effects and that IL-17B is a negative regulator of IL-17E signaling, when they are concomitantly produced and co-expressed in a tissue. Therefore, targeting IL-17B rather than its receptor appears to be a better strategy for anti-cancer therapy. Although the effects of IL-17B neutralization remain to be better defined, the possibility of remodeling the tumor immune microenvironment, in particularly by decreasing the immunosuppression linked to the strong infiltration by macrophages and neutrophils, is an interesting mechanism in the context of resistance to new immunotherapies, such as checkpoint inhibitors and immunogenic chemotherapies, or radiotherapy.

## Author Contributions

JB and NB wrote the manuscript, CD and AD revised the manuscript.

## Conflict of Interest

JB, CD, and AD are employees of OREGA Biotech, JB and NB are shareholders of OREGA Biotech.
